# Muscle MRI characteristic pattern for late-onset TK2 deficiency diagnosis

**DOI:** 10.1007/s00415-021-10957-0

**Published:** 2022-03-14

**Authors:** Cristina Domínguez-González, Roberto Fernández-Torrón, Ursula Moore, Carlos Pablo de Fuenmayor-Fernández de la Hoz, Beatriz Vélez-Gómez, Juan Antonio Cabezas, Jorge Alonso-Pérez, Laura González-Mera, Montse Olivé, Jorge García-García, Germán Moris, Juan Carlos León Hernández, Nuria Muelas, Emilia Servian-Morilla, Miguel A. Martin, Jordi Díaz-Manera, Carmen Paradas

**Affiliations:** 1 Neuromuscular Diseases Unit, Neurology Department, Hospital Universitario 12 de Octubre, Madrid, Spain; 2 Hospital 12 de Octubre Research Institute (imas12), Madrid, Spain; 3 Biomedical Network Research Centre on Rare Diseases (CIBERER), Instituto de Salud Carlos III, Madrid, Spain; 4 Neurology Department, Biodonostia Health Research Institute, Neuromuscular Area, Hospital Donostia, Basque Health Service, Doctor Begiristain, Donostia-San Sebastian, Spain; 5 John Walton Muscular Dystrophy Research Center, University of Newcastle, Newcastle, UK; 6 Neuromuscular Diseases Unit, Neurology Department, Hospital Universitario Virgen del Rocío/ Instituto de Biomedicina de Sevilla, Sevilla, Spain; 7 Centro de Investigación Biomédica en Red Sobre Enfermedades Neurodegenerativas (CIBERNED), Madrid, Spain; 8 Neuromuscular Diseases Unit, Neurology Department, Hospital de la Santa Creu i Sant Pau, Universitat Autònoma de Barcelona, Bellaterra, Spain; 9 Neuromuscular Diseases Unit, Neurology Department, IDIBELL-Hospital Universitari de Bellvitge, Hospitalet de Llobregat, Barcelona, Spain; 10 Institut de Recerca, Hospital de la Santa Creu i Sant Pau, Barcelona, Spain; 11 Department of Neurology. Hospital, Universitario de Albacete., Albacete, Spain; 12 Neuromuscular Diseases Unit. Neurology Department. Hospital, Universitario Central de Asturias, Asturias, Spain; 13 Neurology Department. Hospital, Universitario Nuestra Señora de Candelaria, Tenerife, Spain; 14 Neuromuscular Diseases Unit, Neurology Department, Hospital Universitari i Politecnic La Fe, Neuromuscular and Ataxias Research Group, Instituto de Investigación Sanitaria La Fe, Valencia, Spain; 15 Mitochondrial Disorders Laboratory, Clinical Biochemistry Department, Hospital Universitario 12 de Octubre, Madrid, Spain; 16 Unidad de Enfermedades Neuromusculares (CSUR/EURO-NMD), Hospital Universitario Virgen del Rocío/ Instituto de Biomedicina de Sevilla, Sevilla, Spain; 17 John Walton Muscular Dystrophy Research Centre Newcastle University Translational and Clinical Research Institute Central Parkway, Newcastle upon Tyne, NE1 3BZ UK

**Keywords:** TK2, Mitochondrial myopathy, MRI

## Abstract

**Background and objective:**

TK2 deficiency (TK2d) is a rare mitochondrial disorder that manifests predominantly as a progressive myopathy with a broad spectrum of severity and age of onset. The rate of progression is variable, and the prognosis is poor due to early and severe respiratory involvement. Early and accurate diagnosis is particularly important since a specific treatment is under development. This study aims to evaluate the diagnostic value of lower limb muscle MRI in adult patients with TK2d.

**Methods:**

We studied a cohort of 45 genetically confirmed patients with mitochondrial myopathy (16 with mutations in *TK2*, 9 with mutations in other nuclear genes involved in mitochondrial DNA [mtDNA] synthesis or maintenance, 10 with single mtDNA deletions, and 10 with point mtDNA mutations) to analyze the imaging pattern of fat replacement in lower limb muscles. We compared the identified pattern in patients with TK2d with the MRI pattern of other non-mitochondrial genetic myopathies that share similar clinical characteristics.

**Results:**

We found a consistent lower limb muscle MRI pattern in patients with TK2d characterized by involvement of the *gluteus maximus*, *gastrocnemius medialis*, and *sartorius* muscles. The identified pattern in TK2 patients differs from the known radiological involvement of other resembling muscle dystrophies that share clinical features.

**Conclusions:**

By analyzing the largest cohort of muscle MRI from patients with mitochondrial myopathies studied to date, we identified a characteristic and specific radiological pattern of muscle involvement in patients with TK2d that could be useful to speed up its diagnosis.

**Supplementary Information:**

The online version contains supplementary material available at 10.1007/s00415-021-10957-0.

## Introduction

Recessive mutations in the *TK2* gene lead to an ultra-rare mitochondrial disorder that manifests primarily as a progressive myopathy with early respiratory involvement [1–4]. Mutations in this gene produce a deficiency of the enzyme thymidine kinase 2 (TK2), involved in the intramitochondrial biosynthesis of pyrimidine nucleotides. Consequently, the balance in the nucleotide pool is disturbed, which interferes with the synthesis of the mitochondrial DNA (mtDNA), causing mtDNA depletion or multiple mtDNA deletions [5, 6].

Clinical symptoms of this mitochondrial disorder can start at any age, with a variable rate of progression. The factors that determine the severity of the disease are poorly understood, with the age at onset and the presence of mtDNA depletion the generally accepted prognostic factors [7, 8]. An early infantile-onset with very rapid progression and survival of fewer than 2 years is distinguished as the most severe phenotype, with all these cases being associated with severe muscle mtDNA depletion. Patients with later-onset have a more heterogeneous clinical course with a mean survival of 13–23 years after the onset of initial symptoms [7, 8].

The infantile and childhood-onset are the most frequent presentation, corresponding together to around 80% of patients with TK2 deficiency (TK2d) reported in the literature [7, 8]. Patients who start their symptoms from the second decade of life are the least recognized and are probably underdiagnosed. Taking all the clinical forms together, the main manifestation is a progressive myopathy, which characteristically affects the facial, cervical, axial, bulbar, and respiratory muscles more prominently and, in some cases, it is associated with ptosis and/or ophthalmoparesis, the latter being more frequent in the later-onset type [9]. Other characteristics of TK2d myopathy that can help to differentiate it from other mitochondrial myopathies are the presence of high levels of CK (frequently > 10×) and the existence of dystrophic changes in the muscle biopsy, associated with the common nonspecific signs of mitochondrial dysfunction (ragged-red COX-negative fibers) [3]. However, these features can complicate the diagnosis of TK2d myopathy erroneously leading to the suspicion of a muscular dystrophy.

We have previously analyzed the clinical features of a series of 18 patients with late-onset TK2d, and identified the existence of a possible pattern of involvement in the muscle MRI scans performed in seven of these patients [3]. This pattern was characterized by the predominant involvement of the *gluteus major*, *semitendinosus*, *sartorius*, and *gastrocnemius medialis* muscles in the lower limbs, which was the only examined region.

In this work, we extended the muscle MRI series of the lower limbs of adult patients with TK2d to 16 and compare them with the results of 29 patients with a diagnosis of primary mitochondrial myopathy due to different molecular defects. We also compared the TK2d MRI pattern with that of other genetic myopathies that share some clinical characteristics with TK2d, such as facioscapulohumeral dystrophy (FSHD) due to the weakness of the facial muscles and the oculopharyngeal muscle dystrophy (OPMD) due to the involvement of the ocular and bulbar muscles.

Our objective is to evaluate the diagnostic value of the muscle-imaging pattern to facilitate the identification of adult patients with *TK2* mutations. Early and accurate diagnosis is extremely important in this specific mitochondrial disorder since a very promising therapy is currently under development, which could modify the natural history of the disease [10].

## Methods

### Patients

We recruited patients with the diagnosis of Primary Mitochondrial Myopathy (PMM), defined as an alteration of mitochondrial oxidative phosphorylation of genetic origin that predominantly, but not exclusively, affects the skeletal muscle [11]. Patients came from 9 different Spanish centers: University Hospital 12 de Octubre in Madrid, University Hospital Virgen del Rocío in Seville, University Hospital Juan Ramón Jiménez in Huelva, Hospital de la Santa Creu i Sant Pau and Hospital de Bellvitge in Barcelona, University Hospital of Albacete, Central Hospital of Asturias in Oviedo, Hospital La Candelaria in Tenerife and Hospital Universitari i Politécnic La Fe in Valencia. Clinical data were collected retrospectively from evaluations performed during the routine diagnostic process.

In total, 45 patients were recruited for the study. Twenty-two patients with pathogenic variants in nuclear genes involved in mtDNA synthesis or maintenance were included: 16 patients with recessive mutations in the *TK2* gene, 7 patients with recessive mutations in the *POLG* gene, 1 patient with a heterozygous mutation in the *TWNK* gene, and 1 patient with recessive mutations in the *RRM2B* gene. In addition, 20 patients with primary alterations in mtDNA were also included: 10 patients with a single large-scale deletion in mtDNA and 10 patients with a point mutation in any of the mtDNA genes (4 patients had the m.8344A > G mutation in the *MTTK* gene, 3 patients the m.3243A > G mutation in the *MTTL1* gene and 3 patients had a mutation in the *MTCO1*, *MTTS1*, and *MTTN* genes, respectively). All of them manifested predominantly with muscular symptoms, although not exclusively. The muscle involvement was manifested as chronic external ophthalmoparesis (CPEO) (47%), ptosis (56%), facial weakness (33%), limb muscle weakness (31%), cervical weakness (29%), dysphagia (20%), and respiratory insufficiency (20%). The muscular symptoms and signs of each patient, as well as the molecular diagnosis, are detailed in Table 1.

**Table 1:**
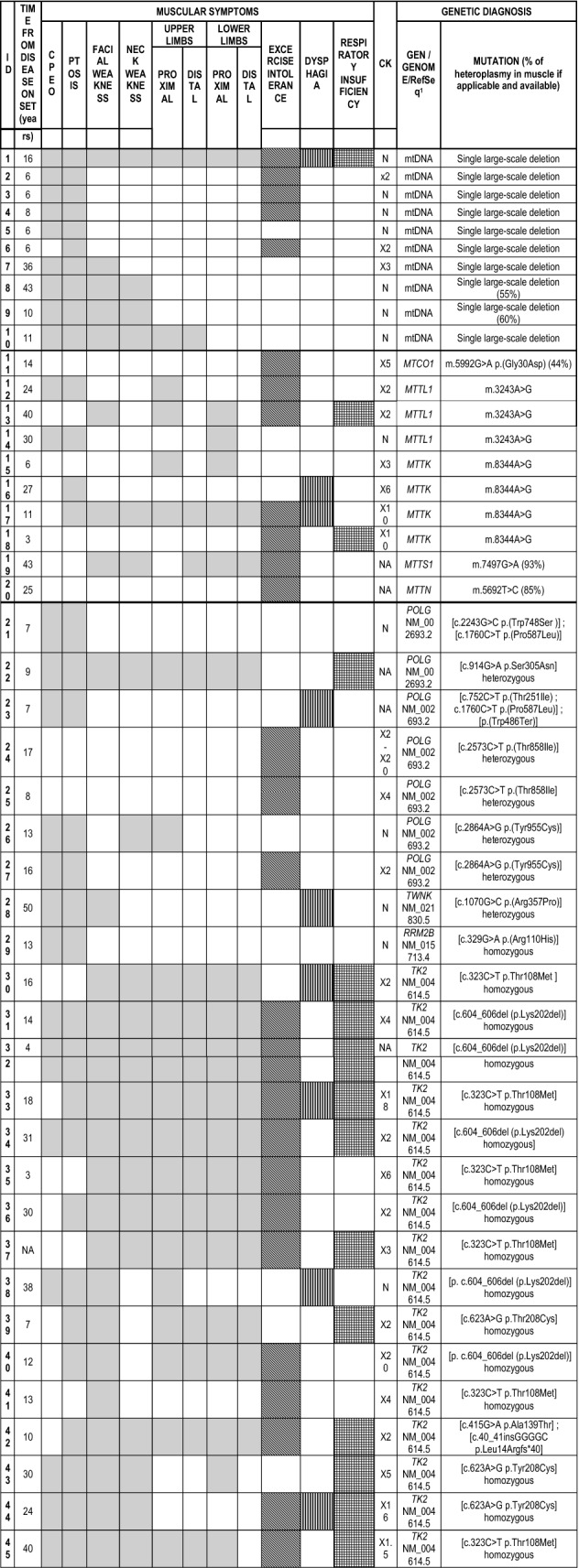
Clinical characteristics summary

The colored cells represent the cases where the symptom or sign is present

*CK* creatine kinase, *NA* not available, *N* normal

^1^mtDNA variants were reference to the GeneBank number NC_012920.1

### Muscle-imaging acquisition and analysis

Muscle MRI studies were performed as part of the routine diagnostic process in a 1.5 T MR scanner at the evaluating institution and were subsequently analyzed and scored by the same neurologist (R F–T), with experience in neuromuscular disorders and muscle imaging. The evaluator was blind to the clinical manifestations and final diagnosis. We analyzed lower limb axial T1-weighted sequences and short-tau inversion recovery (STIR) sequences. Muscles from pelvis, thighs and lower legs were bilaterally scored in axial T1-sequences with the semi-quantitative Mercuri visual scale (MVS) modified by Fisher [12]: 0: normal appearance; 1: mild involvement, less than 30% of individual muscle volume; 2: moderate involvement, 30–60% of individual muscle volumes; 3: severe involvement, > 60% of individual muscle; 4: end stage, all the muscle is severely affected, replaced by increased density of connective tissue and fat, with only a rim of fascia and neurovascular structures distinguishable. Total Mercuri score in a single patient was calculated by adding all values of the Mercuri score of all single muscles.

### Genotype-MRI correlation

We divided the cohort into TK2d patients and patients with other mitochondrial conditions. To establish a pattern of muscle MRI fat replacement in time and correlations to Medical Research Council's scale (MRC scale), hierarchical analysis, and graphical representation as a heatmap was performed using R software, V.3.1.3.

### Muscle MRI in TK2dvs other myopathies

We compared the muscle MRI pattern in TK2d with FSHD and OPMD since they share some clinical characteristics and might be included in the differential diagnosis. Access to muscle MRI raw data from FSHD [13] and OPMD [14] was given by their authors. Boxplots were generated for individual muscles for patients in four groups: (1): TK2d patients, (2) other mitochondrial conditions, (3) FSHD, and (4) OPMD. The right limb was always used. Median MVS in the TK2d group was in turn compared to the median score in FSHD, OPMD, and the other mitochondrial conditions using a Mann–Whitney test for unpaired samples. To demonstrate the key muscles which differ between groups, those muscles showing a significant difference were selected for display.

To compare multiple variables on a similar scale, we used principal component analysis (PCA). Using this approach, we took all Mercuri scores for each muscle selected in each patient and positioned that individual on a 2D chart of principal components, in a way that maximizes the variation between individuals. Each variable, therefore, becomes a vector along which an individual is positioned. Clusters in one area of the chart or following a particular vector, indicate a group of individuals with similar characteristics, while a group with distinct characteristics form a separate cluster, which may be oriented along a different vector line where variability is greatest for this second cluster of individuals. Those muscles showing a significant difference between groups were used to generate a three-principal component analysis. This approach was used to compare the key muscles in TK2d to other mitochondrial diseases, TK2d to FSHD, and TK2d to OPMD. To compare if both FSHD and TK2d groups showed a similar degree of asymmetry, boxplots of individual asymmetry were generated. These were plots showing the difference in Mercuri score between the left and right within each patient individually.

### Statistics

We used the Shapiro–Wilk test to confirm that none of our variables was normally distributed. As such, non-parametric statistic tests were used for the analysis. Kruskal–Wallis test was used to compare quantitative variables across multiple groups while the Wilcoxon test was used for paired comparisons of muscle fat infiltration. The χ^2^ test was used to compare the qualitative variables. To investigate correlations between muscle function tests and MRI findings, Spearman’s rank correlation was used (coefficient reported as *ρ*). The correlation was considered significant if the *p* value was less than 0.05 and *ρ* was 0.6 or higher. Statistical analyses were performed using IBM SPSS Statistics, V.21 (IBM, Armonk, New York, USA).

## Results

### Muscle MRI analysis

All the 45 recruited patients were scanned. The mean age at muscle MRI was 46.4 years (SD 15.8 years) and the meantime from onset to MRI was 18.2 months (SD 12.9 months). In the entire cohort, the *gluteus maximus*, *sartorius*, *gastrocnemius medialis*, *semitendinosus*, *semimembranosus*, and *tensor fascia latae* were the most affected muscles, even though the fat muscle replacement was mild in many cases ((Median Mercuri (MM) 1, range: 0–4). The *popliteal*, *tibialis posterior*, and *flexor digitorum longus* were never or seldomly affected (MM 0).

#### Genotype and muscle MRI pattern

**Fig. 1:**
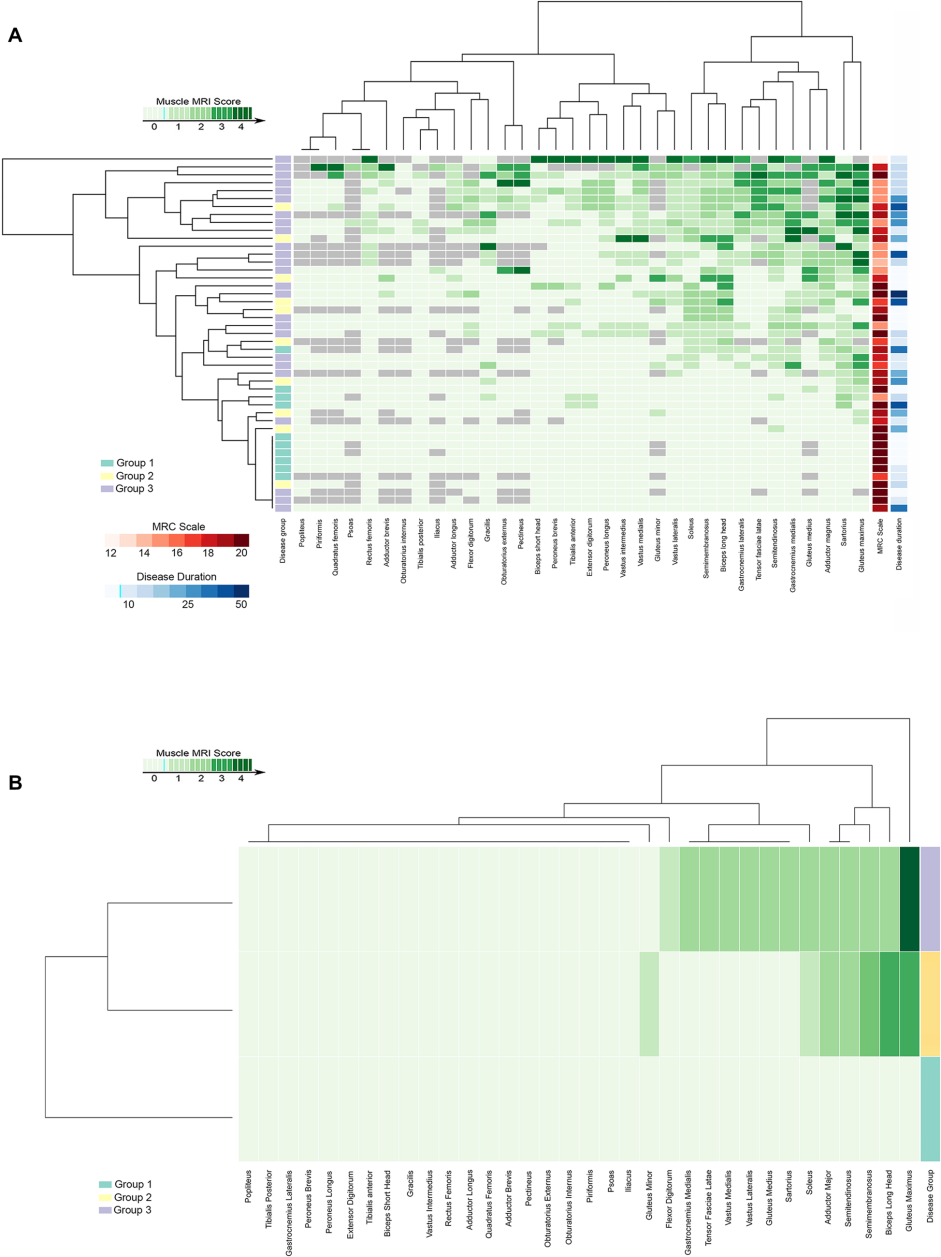
Heatmap of muscles of the lower limbs in patients with mitochondrial myopathies, disease duration, and MRC scale. **A** Heatmap showing involvement of pelvic, thigh and leg muscles. Patients and muscles are ordered according to hierarchical clustering with increasing grading in fat replacement severity from the bottom to the top (patients—rows) and from the left to the right (muscles—columns). The score of a muscle in a patient is indicated by the color of the square. Grey squares mean that data are not available. The column in the top left contains information related to the genetic data of the patient (legend in the bottom left). We have also included a column with information about the muscle strength of the patients measured using the MRC scale in red and a column to the far right with information about the disease duration (time from onset of symptom to the MRI in years). **B** Heatmaps showing the median Mercuri score of the muscles of the pelvis, thigh and leg areas. The three group of diseases and muscles are ordered according to hierarchical clustering. The column in the top right contains information related to the genetic data of the patient (legend in the bottom left). Group 1: mtDNA single large-scale deletion; Group 2: Point mtDNA mutation (*MTCO1*,* MTTL1*,* MTTK*,* MTTS1*, and *MTTN* genes); Group 3: Mutation in nuclear genes involved in mtDNA maintenance (*POLG*, *TWNK*, *RRM2B* and *TK2 genes*). No pattern of muscle involvement was identified in any group

To study if there were different radiological patterns regarding the molecular diagnosis, we divided the cohort into three different groups: Group 1: patients with an mtDNA single large-scale deletion; Group 2: patients with a point mtDNA mutation (*MTCO1*, *MTTL1*, *MTTK*, *MTTS1*, and* MTTN* *genes*); Group 3: patients with mutations in nuclear genes involved in mtDNA synthesis or maintenance (*POLG*,* TWNK*,* RRM2B*, and *TK2*). We found that muscle MRI involvement was more prominent in patients with mutations in nuclear genes (mean total MRC score: 23.6 ± 17.7) than in those with point mtDNA mutation (mean total MRC score = 13.2 ± 12.7) and those with single large-scale mtDNA deletion (mean total MRC score = 2.4 ± 3.5) (Kruskal–Wallis, *p* < 0.001)) (Fig. 1). Thereby, muscle MRI was normal in all but one patient with single mtDNA deletion (group 1) in whom fat replacement signs in *soleus*,* semimembranosus* and long head of the *biceps femoris* were detected. Among the ten patients with point mtDNA mutations (group 2), only three patients had a normal muscle MRI. Nevertheless, and as shown in Fig. 1, the fat replacement pattern was not consistent being *gluteus maximus* (MM 2), long head of *biceps femoris* (MM 2), *semimembranosus* (MM 1.5), and *adductor magnus* (MM 1.5) the most affected muscles. In patients with mutations in nuclear genes (group 3), *gluteus maximus* (MM 3) was the most affected muscle, followed by the *sartorius*, *vastus lateralis*, *vastus medialis*, *tensor fascia latae*, *semimembranosus*, *semitendinosus*, and *gastrocnemius lateralis* (MM 1).

#### Muscle MRI pattern in TK2d

**Fig. 2:**
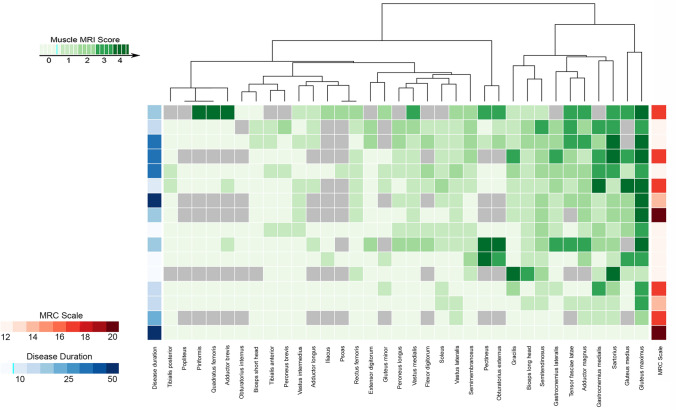
Heatmap of muscles of the lower limbs in patients with *TK2 gene* mutations, disease duration, and MRC scale. Heatmap showing involvement of pelvic, thigh, and leg muscles in patients with mutations in the *TK2* gene. Patients and muscles are ordered according to hierarchical clustering with increasing grading in fat replacement severity from the bottom to the top (patients—rows) and from the left to the right (muscles—columns). The score of a muscle in a patient is indicated by the color of the square. Grey squares mean that data are not available. The column in the top left contains information related to disease duration (time from onset of symptom to the MRI in years) and the column in the top right provides information about muscle strength of the patients measured using the MRC scale in red. Legends can be found at the bottom on the right

**Fig. 3:**
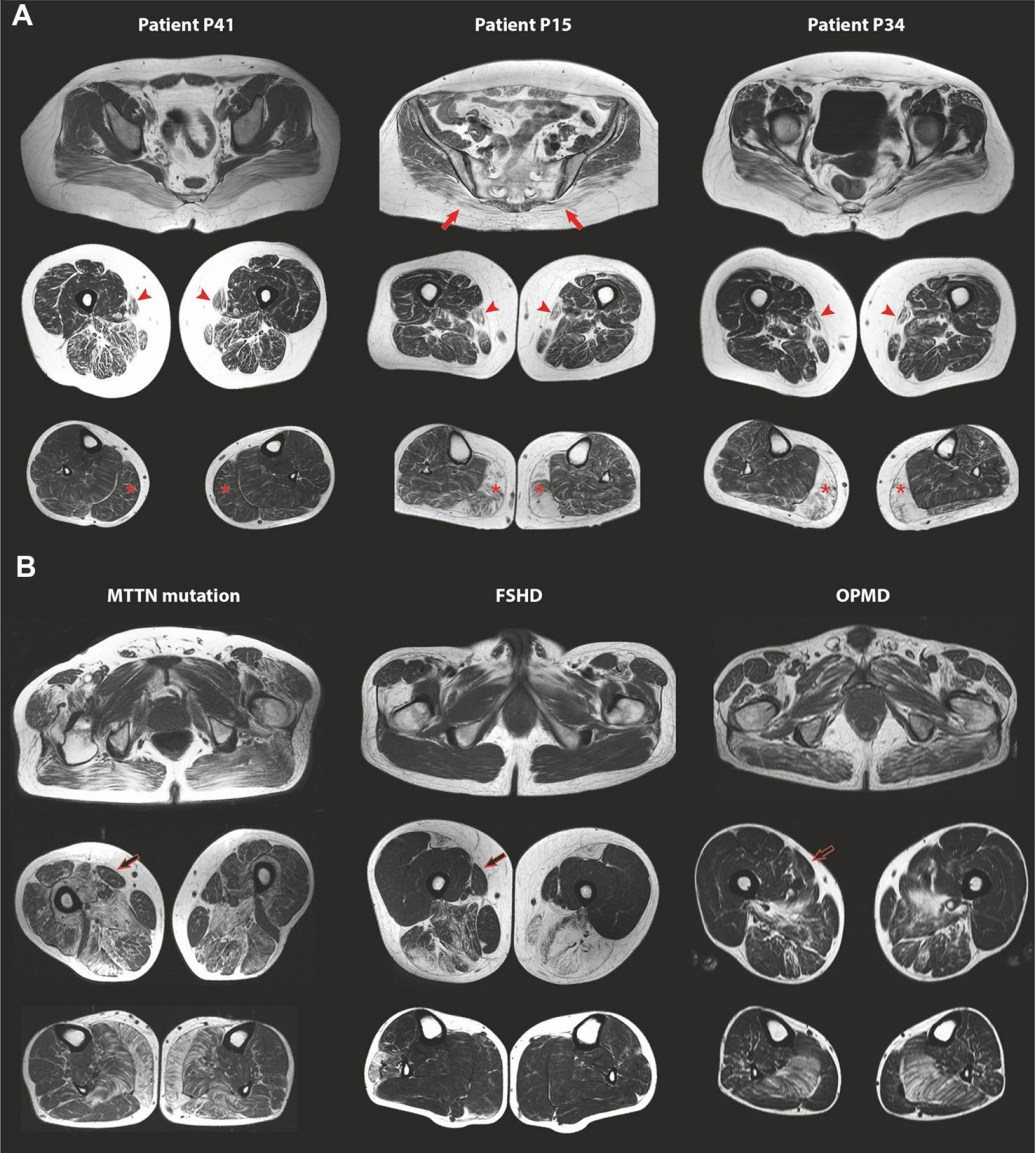
Muscle MRI pattern of the lower limbs in TK2d patients compared to other myopathies. **A** Muscle MRI from three TK2d patients with different disease duration and weakness severity showed that the most affected muscles were the *gluteus maximus* (arrows), the *sartorius* (arrowheads), and the *gastrocnemius medialis* muscles (asterisks). **B** Muscle MRI from patients with other mitochondrial myopathies as the one linked to point mutation in the mitochondrial gene MTTN, or other myopathies with similar clinical characteristics as FSHD or OPMD, showed a very different pattern of muscle involvement, highlighting the full preservation of the *sartorius* muscles (open arrows), even when other muscles are severely degenerated

We focused our attention on the muscle MRI pattern of patients with TK2 deficiency as this is the only mitochondrial myopathy with a specific treatment that can change the natural history of the disease and, therefore, providing a correct diagnosis to the patients is extremely important. *Gluteus maximus* (MM 3), gastrocnemius medialis (MM 2), *gluteus medius*, *sartorius* (MM 2) and *gracilis* (MM 1.5) were the most affected muscles in patients with TK2 mutations. *Gluteus maximus* and *gastrocnemius medialis* were affected in all but one patient. At pelvic level, *gluteus maximus* and *medius* were more affected than *gluteus minor*, while *tensor fascia latae* was moderately affected. In the thighs, the *gracilis*, *sartorius*, *adductor magnus* were the most affected followed by semitendinosus, *semimembranosus*, and long head of *biceps femoris*. Muscles from the anterior compartment of the thigh were less affected. In the legs, the *gastrocnemius medialis*, *gastrocnemius lateralis*, *flexor digitorum*, *soleus*, and *peroneus longus* were affected whereas the *tibialis* anterior was seldomly replaced by fat. We uncovered a consistent MRI pattern in TK2d patients, in which the *gluteus maximus* and *medius*, the *sartorius*, and the *gastrocnemius medialis* were the earliest affected muscles (Figs. 2, 3A, and supplementary Fig. 1).

Correlations between total MRC and muscle MRI were only statistically significant in *adductor major* (R – 0.64, *p* 0.014), *semitendinosus* (R − 0.66, *p* 0.07), *extensor digitorum* (*R* − 0.59, *p* 0.018) (supplementary table 1).

#### TK2d vs FSHD and OPMD MRI pattern comparison

**Fig. 4:**
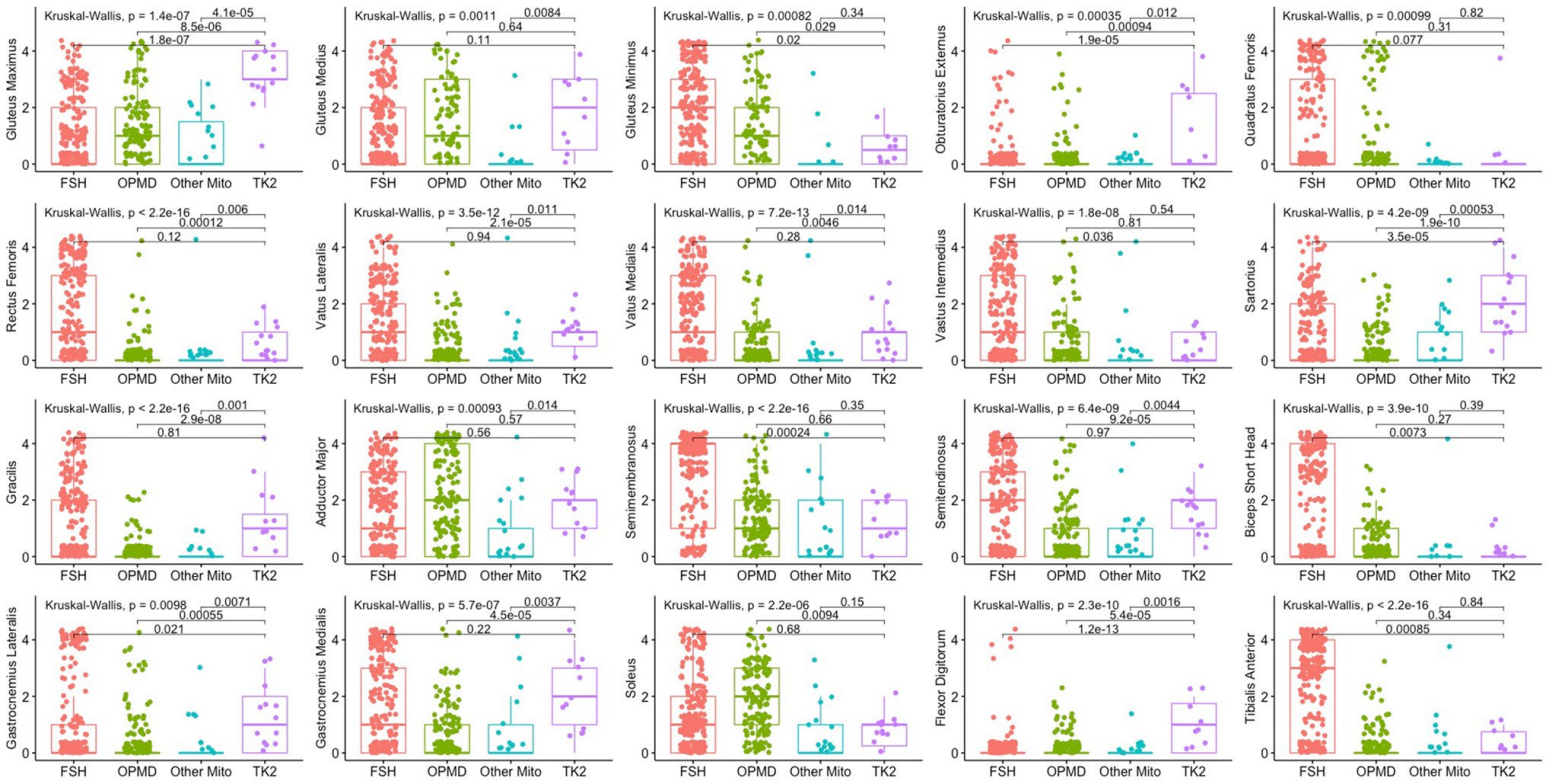
Boxplots showing values of the Mercuri score of muscles of the pelvis, thigh and leg of TK2d, other mitochondrial myopathies, FSHD and OPMD patients. Value of the Kruskal–Wallis test is shown per every comparison and when significant, Wilcoxon test is applied to compare TK2 and the other three diseases

When comparing to other mitochondrial diseases and to other diseases that could resemble clinically, like FSHD and OPMD, muscle MRI from TK2d patients had greater and significant involvement of the *gluteus maximum*, *sartorius*, *gracilis*, *obturator externus*, *gastrocnemius medialis*, and *flexor digitorum longus* muscles (Fig. 3A, B). In contrast, the *rectus femoris*, *semimembranosus*, and *tibialis anterior* muscles were more involved in FSHD (Fig. 4).

**Fig. 5:**
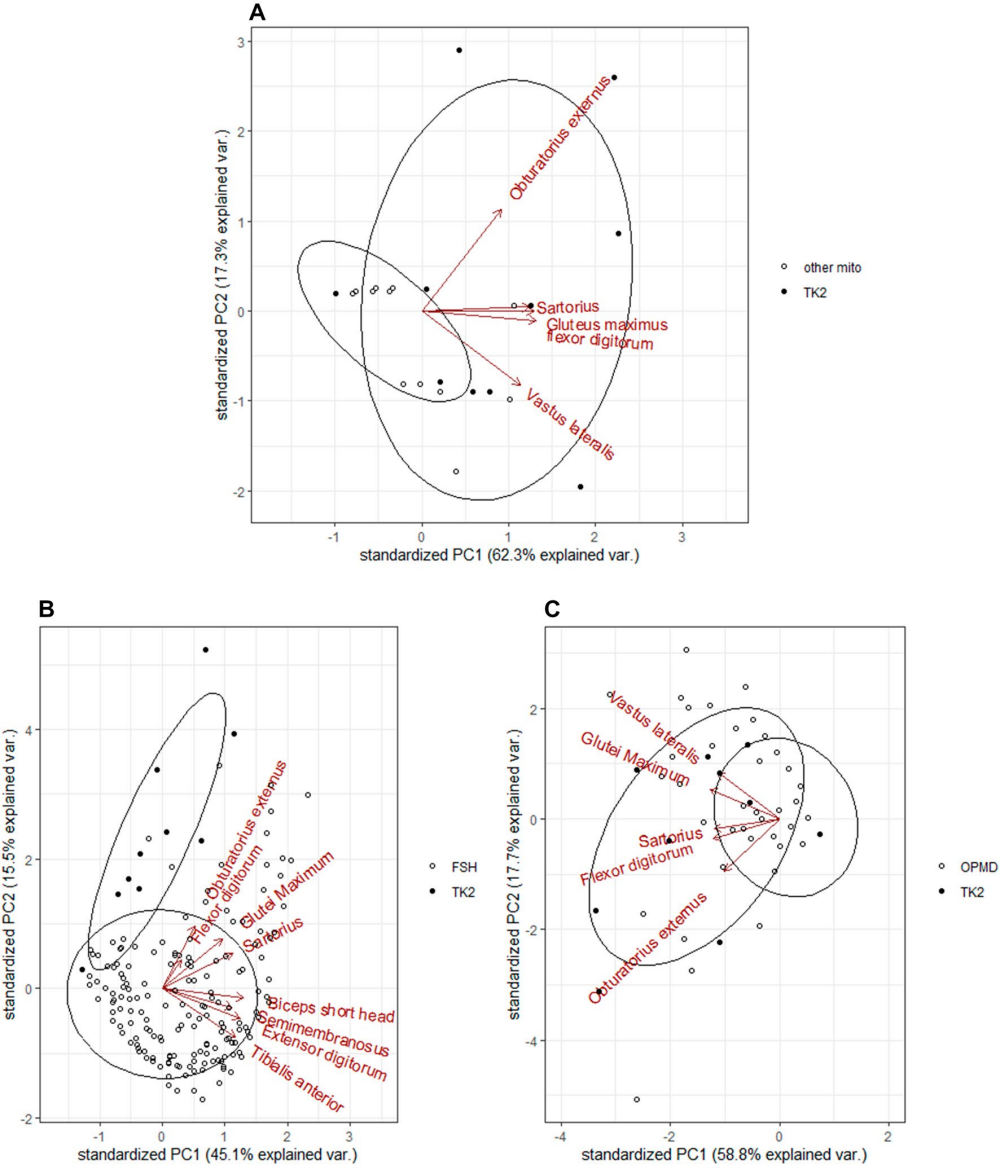
PCA analysis. TK2d compared to other mitochondrial myopathies (**A**) showed that *gluteus maximus*, *vastus lateralis*,* obturator externus*, *sartorius*, and *flexor digitorum* were the muscles that differentiated most one disease to another. **B** TK2d compared to FSHD showed that *gluteus maximus*, *obturator externus*, *sartorius*, the short head of *biceps femoris*, *semimembranosus*, *flexor digitorum*, *extensor digitorum*, and *tibialis anterior* were the muscles that differentiated most one disease to another. **C** TK2d compared to OPMD showed that *gluteus maximus*,* vastus lateralis*, o*bturator externus*, *sartorius*, and *flexor digitorum* were the muscles that differentiated most from one disease to another

When analyzing muscle patterns with PCA, the *gluteus maximum*, *obturator externus*, *vastus lateralis*, *sartorius*, and *flexor digitorum longus* were the most distinctive muscles in TK2d compared to other mitochondrial diseases (Fig. 5A) and OPMD (Fig. 5c). PCA analysis between TK2d and FSHD confirmed that the latter were distinctive muscles in TK2d while short head of *biceps femoris*, *semimembranosus*, *extensor digitorum*, and *tibialis anterior* were those in FSHD (Fig. 5b).

**Fig. 6:**
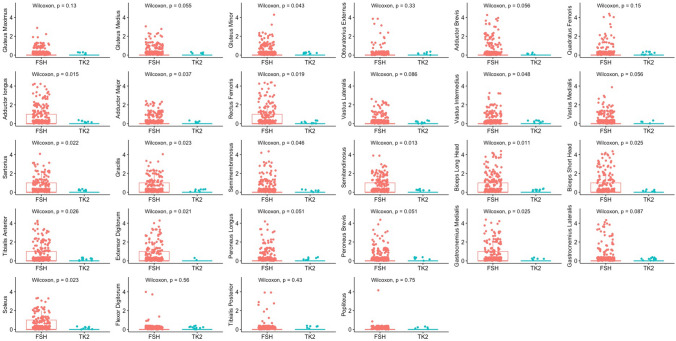
Boxplots showing the difference in Mercuri score between right and left sides of muscles of the pelvis, thigh and leg of FSHD and TK2d. Results of the Wilcoxon test applied to identify significant differences are shown

Muscles from FSHD patients were more asymmetrically involved than TK2d when comparing right and left side muscles individually. This asymmetry was detected especially in *adductor longus*, the long and short head of *biceps femoris*, *rectus femoris*, *gracilis*, and *semitendinosus* in the thigh and *tibialis anterior* and *gastrocnemius medialis* in legs. No degree of asymmetry was detected in the *glutei* (Fig. 6).

## Discussion

Mitochondrial myopathies are a wide group of muscle diseases characterized by heterogeneous clinical manifestations and molecular causes [15, 16]. Reaching a genetic diagnosis is crucial to genetic counseling and precision medicine. Recessive mutations in the *TK2* gene are responsible for a syndrome of mitochondrial depletion/multiple deletions in mtDNA that can manifest at any age, most often as a pure progressive myopathy [2, 4, 7, 8]. In patients with TK2d, oral treatment with thymidine and deoxycytidine nucleosides has shown to prolong survival in the most severe infantile cases and halt the progression of the disease in the other clinical forms [10, 17]. It is, therefore, essential to achieve an early genetic diagnosis in all patients with TK2d. To do so, they must be distinguished from patients with other progressive genetic myopathies, a particularly challenging issue in adult patients, especially in those cases with no prominent ptosis or ophthalmoparesis (25% in our series). From a clinical perspective, the distribution of muscle weakness is critical to suspect a TK2d. Most patients present a combination of facial (with or without ptosis and/or ophthalmoparesis), cervical and axial weakness, with early respiratory insufficiency requiring mechanical ventilation when they are still ambulant [3]. However, in some juvenile-onset patients, the initial diagnostic suspicion may be FSHD, based on prominent facial weakness [18]. On the other hand, some patients have a very late onset with progressive CPEO and dysphagia as the only symptoms for many years, and they can, therefore, be confused with OPMD.

In a previous work, we described the muscle involvement in the MRI of the lower limbs of seven patients with TK2d, suggesting a characteristic pattern with potential diagnostic utility [3]. In the present study, our results confirm the consistency of this pattern in a larger cohort of patients and demonstrate its specificity when compared with the pattern of other myopathies, mimicking the clinical presentation of TK2d, whether of mitochondrial origin or not.

In this study, we demonstrated that among patients with PMM, muscle fat replacement in the lower limbs is heterogeneous in patients with mtDNA mutations, without a specific pattern that distinguishes them as a group. Furthermore, in the majority of patients harboring a single large-scale mtDNA deletion in our cohort, muscle MRI was normal. In the group of patients with nuclear gene mutations, we also did not identify a characteristic pattern that allows us to differentiate them as a group. However, a hierarchical analysis identified that *gluteus maximus*, *gluteus medius*, *sartorius*, *gracilis*, and *gastrocnemius medialis* were the most affected muscles in patients with TK2d, compared to mutations in the other nuclear genes, this pattern being the only one identified in patients with PMM. Our results also indicated that *gluteus maximus*, *gastrocnemius medialis*, and *sartorius* were the earliest affected muscles in patients with *TK2* mutations. The s*artorius* is a muscle that is not usually involved or is even preserved until late stages in many myopathies, so this finding can help in the differential diagnosis. Early involvement of the *sartorius* muscle has also been described in patients with mutations in *SPEN1* [19], *RYR1* [20], *TNPO3* [21], and *MYH7 *[22], in some congenital myopathies like *ACTA1* [23], and in myofibrillar myopathies with mutations in *DES* [24] or *CRYAB* [12].

We have applied a PCA test to the analysis of T1w imaging data quantified using Mercuri score of two large cohorts of patients with FSHD and OPMD patients already published [13, 14] and have included the results of this cohort of TK2d patients in the analysis, with the aim to (1) validate if there is a different pattern of involvement and (2) identify which muscles can be useful to differentiate one disease from the other. We have observed that *gluteus maximus*, *obturator externus*, *vastus lateralis*, *sartorius*, and *flexor digitorum longus* differentiate TK2 myopathy from FSHD and OPMD. In FSHD, involvement of the posterior compartment of the thigh is a common finding, although the most frequently and severely involved muscle is the *semimembranosus*, while in legs the most affected muscles are *tibialis anterior*, *soleus*, and *gastrocnemius medialis*. In addition, muscle MRI involvement in FSHD is commonly asymmetric, while it is symmetric in TK2d as shown here in Fig. 6 [13]. The muscle MRI pattern of OPMD also differs from TK2d, showing in the pelvis a progressive involvement of *glutei muscles*, especially *gluteus minimus*, in the thighs a preferential involvement of posterior muscles and the *adductor* muscles, and in the legs, an early involvement of the *soleus* and *peroneus* muscles [14].

Muscle MRI is a complementary tool used to detect patterns of muscle fat replacement that could help in the diagnosis of patients with myopathies. Although next-generation sequencing is becoming widely accessible in several centers, many of the mitochondrial genes are not included in the panels commercially available, and in several countries, the diagnosis continues to be based on Sanger sequencing. In addition, interpretation of NGS results is not always straightforward and the addition of clinical, muscle biopsy, and MRI data can be helpful. In this sense, patterns of muscle MRI infiltration have been described for many diseases [25, 26] and there is an increasing body of evidence demonstrating that MRI-based artificial intelligence tools could accurately diagnose muscular disorders [27]. Moreover, muscle fat replacement has been shown to correlate with results of muscle function tests, and the value of MRI as a possible biomarker to evaluate response to treatments in clinical trials is increasingly being recognized [28, 29].

Despite all these advantages, there are not many studies of muscle MRI in mitochondrial myopathies [30, 31]. The largest series published to date included 16 patients with mitochondrial myopathy due to different mutations only in the mtDNA (including single large-scale deletions and point mtDNA mutations [32]) and a characteristic pattern of muscle involvement was not identified.

In this study, we have used conventional qualitative MRI imaging techniques, such as T1w, to identify fat replacement in muscles. However, in the last 10 years, there has been a notorious development of quantitative sequences such as Dixon or water T2 that can quantify fat replacement and water content, respectively, and that have been validated as good candidate imaging biomarkers to follow-up patients with neuromuscular diseases. These sequences have not been used to study patients with mitochondrial myopathies [32]. Phosphorus magnetic resonance spectroscopy (^31^P-MRS) could be of great interest in this field as it monitors mitochondrial function in vivo in skeletal muscle [33, 34]. In fact, ^31^P-MRS of resting muscle is sensitive and specific for diagnosing mitochondrial myopathies [35] and has been used in small studies to evaluate therapeutic interventions [36]. However, ^31^P-MRS is a complex technique not available in many centers and, therefore, not included in the standards of care for mitochondrial diseases [37].

There are some limitations to this study. The cohort of patients with other mitochondrial myopathies is genetically very heterogeneous and, therefore, it is expected that their muscle MRI are different. Another limitation is the use of a semi-quantitative scoring method. Although the method is commonly used in other studies, inter-interpreter variability is intrinsic to this type of rating scale.

In conclusion, analyzing the largest cohort of muscle-imaging studies from patients with mitochondrial myopathies to date, we have identified a characteristic and specific radiological pattern of muscle involvement of the lower limbs in patients with TK2d. This pattern allows: (1) to distinguish TK2d from other mitochondrial myopathies and, therefore, to guide genetic testing, (2) to support the diagnosis when the identified variants in the *TK2* gene are classified as of uncertain clinical significance, and (3) to differentiate TK2d from other myopathies with similar clinical manifestations, such as FSHD and OPMD, which is particularly important now because genetic studies are usually prioritized over muscle biopsies during the diagnostic process.

## Supplementary Information

Below is the link to the electronic supplementary material.

Supplementary file1 (DOCX 28 KB)

Supplementary file2 (DOCX 836 KB)

## Data Availability

Data are available upon reasonable request. The data central to this study are available on request from suitably qualified researchers.

## Declarations

### Provenance and peer review

Not commissioned; externally peer-reviewed.
