# Supplementary material for: Muscle MRI characteristic pattern for late-onset TK2 deficiency diagnosis
Source: J Neurol. 2022 Mar 14;269(7):3550–62. doi: 10.1007/s00415-021-10957-0 (PMC9217784; doi:10.1007/s00415-021-10957-0)
Supplement: Supplementary file 1 — Supplementary file1 (DOCX 28 KB) [file 415_2021_10957_MOESM1_ESM.docx]

|  | TOTALMRC | | | Time from onset to MRI (years) | | |
| --- | --- | --- | --- | --- | --- | --- |
|  | Correlationcoefficient | Sig. (bilateral) | N | Correlationcoefficient | Sig. (bilateral) | N |
| GluteiMaximumR | -,308 | ,263 | 15 | ,169 | ,547 | 15 |
| GluteiMaximumL | -,269 | ,333 | 15 | ,108 | ,702 | 15 |
| GluteiMediusR | -,256 | ,423 | 12 | ,014 | ,965 | 12 |
| GluteiMediusL | -,256 | ,423 | 12 | ,014 | ,965 | 12 |
| GluteiMinorR | -,246 | ,493 | 10 | -,040 | ,912 | 10 |
| GluteiMinorL | -,324 | ,361 | 10 | -,104 | ,774 | 10 |
| Tensor fasciaelataeR | -,318 | ,289 | 13 | ,327 | ,276 | 13 |
| Tensor fasciaelataeL | -,356 | ,233 | 13 | ,511 | ,074 | 13 |
| ObturatoriusinternusR | -,541 | ,107 | 10 | ,174 | ,631 | 10 |
| ObturatoriusinternusL | -,541 | ,107 | 10 | ,174 | ,631 | 10 |
| ObturatoriusexternusR | -,504 | ,114 | 11 | ,114 | ,738 | 11 |
| ObturatoriusexternusL | -,504 | ,114 | 11 | ,114 | ,738 | 11 |
| PectineusR | -,455 | ,159 | 11 | -,026 | ,939 | 11 |
| PectineusL | -,455 | ,159 | 11 | -,026 | ,939 | 11 |
| AdductorbrevisR | ,046 | ,894 | 11 | ,017 | ,959 | 11 |
| AdductorbrevisL | -,056 | ,870 | 11 | ,021 | ,950 | 11 |
| AdductorlongusR | -,243 | ,472 | 11 | ,173 | ,611 | 11 |
| AdductorlongusL | -,147 | ,665 | 11 | ,178 | ,600 | 11 |
| AdductormajorR | -,545^*^ | ,044 | 14 | -,046 | ,876 | 14 |
| AdductormajorL | -,640^*^ | ,014 | 14 | -,163 | ,577 | 14 |
| RectusfemorisR | -,022 | ,937 | 15 | ,155 | ,581 | 15 |
| RectusfemorisL | -,060 | ,831 | 15 | -,019 | ,947 | 15 |
| VastuslateralisR | -,054 | ,849 | 15 | ,351 | ,199 | 15 |
| VastuslateralisL | -,150 | ,595 | 15 | ,283 | ,306 | 15 |
| VastusintermediusR | -,138 | ,623 | 15 | ,016 | ,954 | 15 |
| VastusintermediusL | -,022 | ,937 | 15 | ,085 | ,764 | 15 |
| VastusmedialisR | -,397 | ,143 | 15 | ,207 | ,460 | 15 |
| VastusmedialisL | -,265 | ,339 | 15 | ,134 | ,634 | 15 |
| SartoriusR | -,350 | ,201 | 15 | ,184 | ,511 | 15 |
| SartoriusL | -,229 | ,412 | 15 | ,173 | ,537 | 15 |
| GracillisR | -,087 | ,757 | 15 | ,104 | ,713 | 15 |
| GracillisL | -,116 | ,679 | 15 | ,135 | ,632 | 15 |
| SemimembranosusR | -,317 | ,249 | 15 | -,287 | ,300 | 15 |
| SemimembranosusL | -,414 | ,125 | 15 | -,370 | ,175 | 15 |
| SemitendinosusR | -,664^**^ | ,007 | 15 | -,067 | ,813 | 15 |
| SemitendinosusL | -,521^*^ | ,046 | 15 | ,080 | ,777 | 15 |
| BicepslongheadR | -,591^*^ | ,020 | 15 | -,208 | ,457 | 15 |
| BicepslongheadL | -,154 | ,583 | 15 | -,218 | ,435 | 15 |
| Biceps short headR | -,317 | ,269 | 14 | ,076 | ,796 | 14 |
| Biceps short headL | -,180 | ,538 | 14 | ,043 | ,883 | 14 |
| Tibialisanterior | -,501 | ,057 | 15 | ,059 | ,841 | 14 |
| TibialisanteriorL | -,433 | ,107 | 15 | ,295 | ,307 | 14 |
| Extensor digitorumR | -,599^*^ | ,018 | 15 | ,130 | ,659 | 14 |
| Extensor digitorumL | -,575^*^ | ,025 | 15 | ,133 | ,649 | 14 |
| PeroneuslongusR | -,611^*^ | ,015 | 15 | ,353 | ,216 | 14 |
| PeroneuslongusL | -,494 | ,061 | 15 | ,186 | ,525 | 14 |
| PeroneusbrevisR | -,243 | ,383 | 15 | -,064 | ,827 | 14 |
| PeroneusbrevisL | -,243 | ,383 | 15 | -,064 | ,827 | 14 |
| GastrocnemiusmedialisR | -,101 | ,720 | 15 | -,026 | ,930 | 14 |
| GastrocnemiusmedialisL | -,101 | ,720 | 15 | -,026 | ,930 | 14 |
| GastrocnemiuslateralisR | -,398 | ,141 | 15 | ,261 | ,367 | 14 |
| GastrocnemiuslaterallisL | -,339 | ,217 | 15 | ,222 | ,445 | 14 |
| SoleusR | -,212 | ,449 | 15 | ,044 | ,881 | 14 |
| SoleusL | -,228 | ,413 | 15 | ,020 | ,946 | 14 |
| Flexor digitorumR | -,635^*^ | ,036 | 11 | ,379 | ,280 | 10 |
| Flexor digitorumL | -,481 | ,114 | 12 | ,324 | ,332 | 11 |

**Supplementary table . Correlation between individual Mercuri visual score per muscle, time from onset and total strength.**Spearman´s correlation values between individual muscles (R: right, L: left), total MRC strength and time from onset to scan. In those muscles with *, correlation is significant 0,05 (bilateral), whereas ** correlation coeficient is significant at 0,01 (bilateral).
