# Supplementary material for: Muscle MRI characteristic pattern for late-onset TK2 deficiency diagnosis
Source: J Neurol. 2022 Mar 14;269(7):3550–62. doi: 10.1007/s00415-021-10957-0 (PMC9217784; doi:10.1007/s00415-021-10957-0)
Supplement: Supplementary file 2 — Supplementary file2 (DOCX 836 KB) [file 415_2021_10957_MOESM2_ESM.docx]

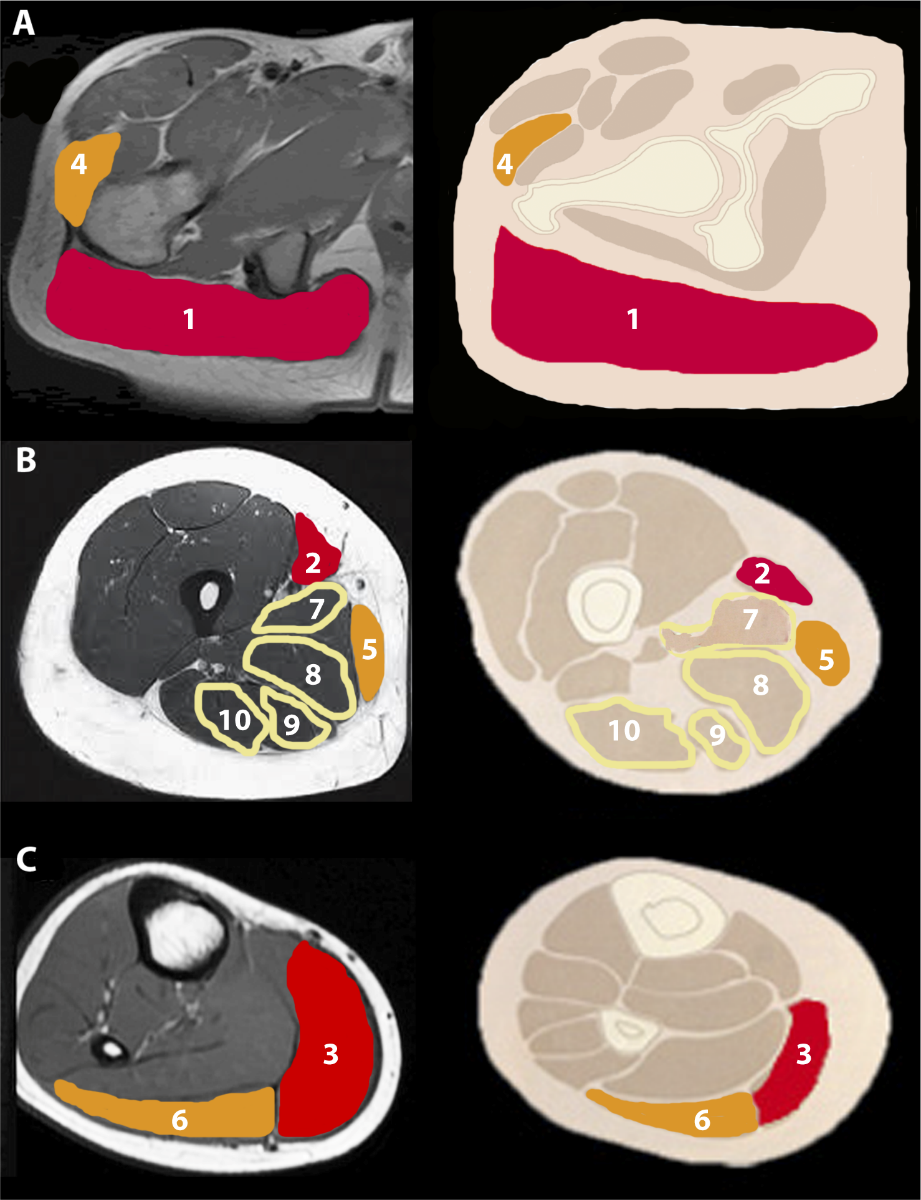


**Supplementary figure 1.** The figure shows the evolution of degeneration in muscles at the level of hip (A), thigh (B) and lower leg (C) associated to mutations in the *TK2* gene. The muscle in red are the earliest affected muscles (the gluteus maximus at hip [1], the sartorius at thigh [2] and the gastrocnemius medialis at lower leg [3]), followed by muscles in orange at later stages (the gluteus medius at hip [4], the gracilis at thigh [5], and the gastrocnemius lateralis at lower leg [6]. Muscles outlines in yellow are less frequently affected (the adductor magnus [7], semimembranosus [8], semitendinosus [9], and long head of biceps femoris at thigh [10]).
